# Motor imagery enhances performance beyond the imagined action

**DOI:** 10.1073/pnas.2423642122

**Published:** 2025-05-13

**Authors:** Magdalena Gippert, Pei-Cheng Shih, Tobias Heed, Ian S. Howard, Mina Jamshidi Idaji, Arno Villringer, Bernhard Sehm, Vadim V. Nikulin

**Affiliations:** ^a^Department of Neurology, Max Planck Institute for Human Cognitive and Brain Sciences, Leipzig 04103, Germany; ^b^Sony Computer Science Laboratories, Tokyo 141-0022, Japan; ^c^Department of Psychology and Centre for Cognitive Neuroscience, University of Salzburg, Salzburg 5020, Austria; ^d^School of Engineering, Computing and Mathematics, Faculty of Science and Engineering, University of Plymouth, Plymouth PL4 8AA, United Kingdom; ^e^Machine Learning Group, Berlin Institute for the Foundations of Learning and Data, Berlin 10587, Germany; ^f^Machine Learning Group, Institute of Software Engineering and Theoretical Computer Science, Electrical Engineering and Computer Science Faculty, Technical University Berlin, Berlin 10587, Germany; ^g^Department of Neurology, Martin Luther University of Halle-Wittenberg, Halle (Saale) 06120, Germany

**Keywords:** motor learning, motor imagery, adaptation, force field, motor sequence

## Abstract

Motor imagery is frequently utilized to improve the performance of specific target movements in sports and rehabilitation. In this study, we show that motor imagery can facilitate learning of not only the imagined target movements but also sequentially linked overt movements. Hybrid sequences comprising imagined and physically executed segments allowed participants to learn specific movement characteristics of the executed segments when they were consistently associated with specific imagined segments. Electrophysiological recordings revealed that the degree of event-related synchronization in the alpha and beta bands during a basic motor imagery task was correlated with imagery-evoked motor learning. Thus, both behavioral and neural evidence indicate that motor imagery’s benefits extend beyond the imagined movements, improving performance in linked overt movements. This provides decisive evidence for the functional equivalence of imagined and overt movements and suggests applications for imagery in sports and rehabilitation.

## Significance

Specific cueing movements embedded within a sequence can influence execution and learning of individual segments. We investigated whether the facilitative effect of linked prior movements could also be achieved by imagining the prior movements. Indeed, motor imagery of prior reaching movements allowed motor learning in an established motor adaptation paradigm. Our results go beyond a simple demonstration that motor imagery resembles performance of an actual movement in the brain by showing that imagined movements can also enhance performance of different linked movements. In addition, we show that the strength of neural activity during a basic motor imagery task is associated with motor adaptation. Our results indicate effective use cases of motor imagery in sports and rehabilitation.

Motor imagery refers to the mental simulation, or rehearsal, of movement without physical execution. Executed and imagined movements exhibit strong similarities in timing (1), associated cardiac and respiratory activity (2, 3), activated brain structures (4) and neural responses (5). This suggests shared mechanisms between overt and imagined movements (6, 7). In addition, motor imagery supports learning during laboratory motor tasks such as sequence learning (8), strength (9) and balance tasks (10); in rehabilitation after neural (11) and musculoskeletal injury (12); and in sports performance improvement interventions (13). Even motor adaptation, the gradual adjustment of movements to changed environmental dynamics, benefits from motor imagery (14). Thus, motor imagery is an effective intervention for performance improvement across a wide variety of movement-related contexts (for meta-analysis, see ref. 15; for reviews, see refs. 16 and 17).

Functional MRI (fMRI) and magnetoencephalography revealed overlapping activation in motor-related regions—including premotor cortices, supplementary motor area, parietal regions, and cerebellar networks—during motor imagery and executed movements, supporting the concept of shared neural substrates (18–20). Training interventions demonstrated that motor imagery induces cortical plasticity comparable to physical practice (18, 21), with an fMRI study showing shifts in the cerebellum and sensorimotor cortex activity after multiday training (22). In addition, electrophysiological studies have shown similar oscillatory dynamics over sensorimotor areas comparing imagined and executed movements (5, 23).

Taken together, studies on motor imagery have typically investigated the behavioral and brain responses to the mental rehearsal of a specific target movement (4, 24, 25). However, movements in daily life are usually organized in motor sequences that comprise multiple, individual movements. In sports, for example, preparatory movements are not necessarily biomechanically advantageous but rather prepare the athlete for a subsequent, specific muscle activation, rhythm, or timing. For instance, think of a basketball player bouncing the ball in a particular way or a particular number of times before a free throw. Such trained motor sequences support motor learning by cueing the corresponding target movement. Evidence suggests that the brain stores representations of entire motor sequences (26) and that individual motor segments affect the execution of the linked segments that follow (27). For example, letters are handwritten slightly differently depending on the preceding letter.

Motor adaptation refers to the process by which the motor system adjusts its output to compensate for changes in the environment or the body. When participants experience two randomly occurring perturbations during a reaching movement, e.g., in an interference force field task, they do not exhibit adaptation, given the perturbations’ randomness. Yet, if each perturbation is associated with a unique prior movement, participants can successfully adapt their movements (28). This kind of learning relies on associations between the kinematics of the prior movement and the adjustments required for the target movement, that is, the perturbed reach. The smaller the variability of the associated prior movement, the stronger the participants adapt (29). Thus, the execution of a perturbed target movement benefits from a specific, regularly preceding movement in the sequence. Given the importance of sequence storage for learning and execution of linked overt movements, we asked whether a similar mechanism is at play when prior movements of a sequence are imagined rather than executed. Previous studies proposed that overt movement and motor imagery are functionally equivalent (7, 24, 30, 31). This suggests that overt segments in a motor sequence can be substituted with motor imagery without altering the overall outcome of the sequence. To comprehensively test this hypothesis, we integrated either executed or imagined prior movements with a perturbed overt target reach within a single sequence in an interference force field task. This paradigm enabled us to utilize reaching adaptation to study motor imagery, a movement with a fundamental role in daily activities, while also allowing for precise measurement of kinematic parameters to provide quantitative data for comparing the consequences of imagined and executed prior reaches. Participants successfully adapted their reaching movements to the interfering perturbations both when the linked, prior movement was executed or imagined. However, the adaptation was reduced for the imagined group. Moreover, power changes in the alpha and beta band during an independent basic motor imagery task, such as imagined fist clenching, were correlated with adaptation performance in the interference force field reaching task.

## Results

**Fig. 1.**
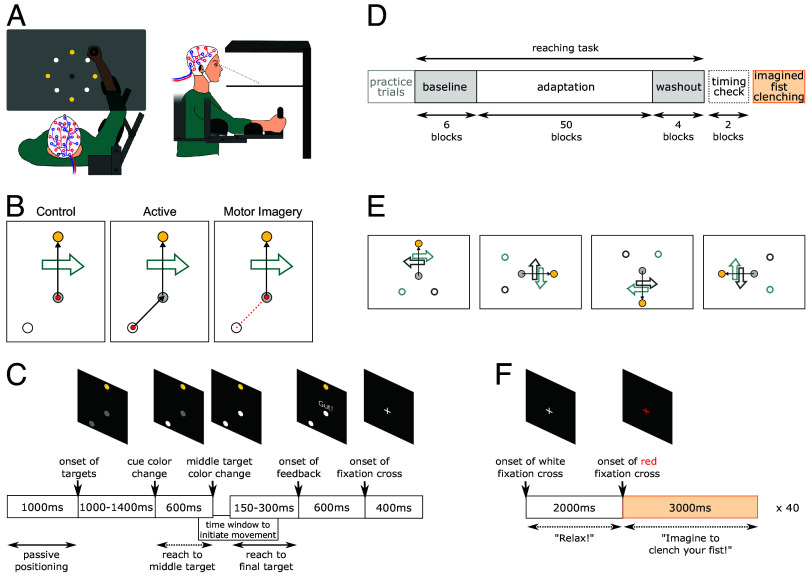
Experimental design and protocol. (*A*) Kinarm Exoskeleton Robot Lab. The screen/mirror is displayed transparent here for illustration; however, in the experiment participants were not able to see their arms. (*B*) Exemplary trial of the reaching task separately for each experimental group. White dot—cue, gray dot—middle target, yellow dot—final target, red dot—hand position at the beginning of a trial, black arrow—instructed reaching path during the trial, red arrow—imaginary reaching path, big teal arrow—exemplary force field direction. (*C*) Reaching task trial sequence. The prior movement, that is, the reach from cue to middle target was only overtly performed by the active group. The imagery group only imagined the prior movement while the hand was already positioned at the middle target. The control group did not perform or imagine a prior movement at all. Feedback about the movement time between middle and final target was given after every trial to encourage a similar reaching speed across participants. (*D*) Experimental phases. The timing check task consisted of active reaches regardless of group membership. The imagined fist clenching task was only performed by participants in the MI group. (*E*) Exemplary cue/final target combinations with big arrows representing force field direction depending on the cue’s location. White dot with teal/gray border—cue, gray dot—middle target, yellow dot—final target, black arrow—desired reaching path, and big teal/gray arrow—force field direction. (*F*) Imagined fist clenching task sequence. Participants were instructed to relax or to perform kinesthetic motor imagery of clenching their fist around the Kinarm’s handle when the fixation cross turned white or red, respectively. (*A*–*E*) were adapted from 32.

60 participants made reaching movements to different targets in an Exoskeleton Lab (Kinarm, Kingston Ontario). This device allows administration of precise perturbations of arm movements (Fig. 1*A*). Participants were randomly assigned to one of three different groups (20 participants per group; see Fig. 1*B*). Participants of the control group made a single reach from a middle to a final target. The position of an additional cue target indicated the possible perturbation direction during each trial. Participants of the active group performed a movement sequence that started with a reach from the cue’s position to the middle target, followed by a reach from the middle to the final target. Participants of the motor imagery (MI) group initially positioned their hand at the middle target but were instructed to perform kinesthetic imagery of a movement from the cue to the middle target and to subsequently execute the reach from the middle to the final target. In this way they performed a hybrid sequence that encompassed both an imagined and an executed segment. The start signal for each (imagined or overt) reaching movement was a color change of the cue and middle target, respectively (Fig. 1*C*). The reaching task was performed in blocks: 6 baseline, 50 adaptation, and 4 washout (Fig. 1*D*). Each block comprised 18 trials. In the baseline phase, reaches were not perturbed. During the adaptation phase, a velocity-dependent, curl force field perturbed the reach between the middle and final targets. The direction of the force field was associated with the location of the cue in relation to the final target and therefore the direction of the (imagined) prior movement in the active and MI groups (Fig. 1*E*). No group was informed of this relation and, according to posttask questioning, participants were not explicitly aware of the association between the force field direction and the cues’ location. In the washout phase, reaches were no longer perturbed. This allowed measurement of aftereffects induced by the adaptation procedure.

### Kinematic Results.

**Fig. 2.**
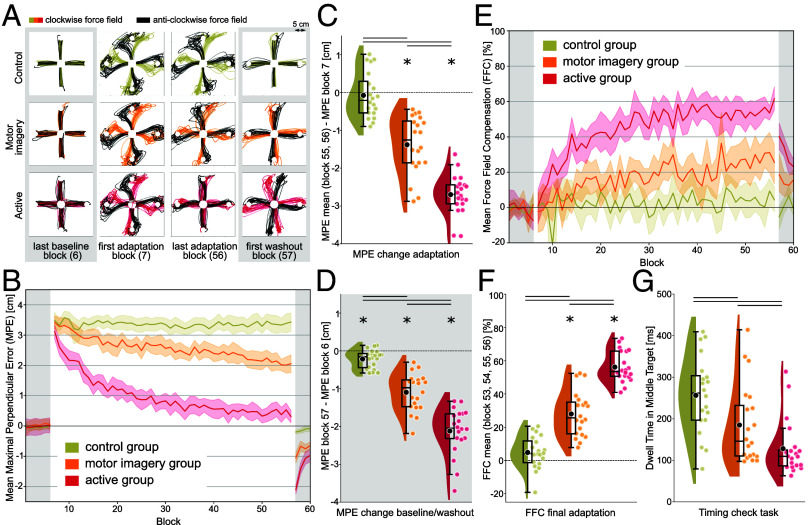
Behavioral results. Data of the Control and Active groups were also reported in ref. 32. (*A*) Reaching trajectories. Single trial trajectories from middle to final targets of all participants (N = 20 in each group) in specific blocks of the experiment. For each final target/cue position combination, one reaching trajectory per participant per block is shown. Force fields were only present in adaptation blocks. (*B*) Averaged maximal perpendicular error (MPE) over trials and participants for each block. Force fields were present from block 7 to block 56 (adaptation blocks = white background). Error bands indicate 95% CIs. (*C*) Change of MPE from beginning to end of adaptation. Each colored dot depicts the difference between average performance in the first and last two adaptation blocks of one participant. Black dots mark the respective group averages. Lines denote significant differences between groups *P* < 0.05. Stars mark significant within-group effects *P* < 0.05. (*D*) Change of MPE from baseline to washout. The differences between the first washout and the last baseline block are displayed per participant and as a group average. (*E*) Averaged FFC over trials and participants for each block. (*F*) Average FFC in the last 4 adaptation blocks. (*G*) Comparison of median dwell time in the middle target in the timing check task. Each colored dot depicts the median dwell time of one participant.

Reaching trajectories were curved in the direction of the force fields at the beginning of the adaptation phase (Fig. 2*A*). The active group adjusted their reaches to counteract the forces by the end of this phase, while the control group’s reaches remained strongly perturbed. This result pattern replicates previous findings and confirms that participants can adapt to multiple environmental perturbations when these are disambiguated by prior segments in a longer movement sequence (e.g., ref. 28). The key finding is that, like the active group, the MI group exhibited adaptation. However, the adaptation was weaker, with significant curvature of reaches remaining even after all 50 blocks of the adaptation phase. Thus, adaptation of the imagery group appeared to be intermediate between the active and control groups. Similarly, aftereffects in the washout phase, manifested by deflections of reaches in the opposite direction of the previously experienced force fields, were strong in the active group, less strong for MI, and virtually absent in the controls.

To systematically quantify the extent of adaptation, we calculated the maximal signed deviation from a straight line between the middle and final targets for each reach. This Maximal Perpendicular Error (MPE) was then averaged over trials within each block (Fig. 2*B*). We compared the MPE at the beginning and end of the adaptation phase within and across groups. Paired *t* tests within each group revealed that MPE change during the adaptation phase was significant in the active (*t*(19) = 22.87, *P* = 1.3e-14; all reported *P*-values for *t* tests are corrected for multiple comparisons) and MI groups (*t*(19) = 8.01, *P* = 6.5e-07) but not in the control group (*t*(19) = 0.54, *P* = 0.59). Active and MI participants exhibited significantly reduced error at the end of the adaptation phase. Still, *t* tests between groups were significant for all three pair-wise comparisons (*t*(18) = −14.60, *P*_active/control_ = 2.2e-16; *t*(18) = −6.32, *P*_active/MI_ = 6.3e-07; *t*(18) = −5.96, *P*_MI/control_ = 1.3e-06). This confirms that MPE change was strongest in the active group but that MI participants also performed better than control group participants (Fig. 2*C*). Error reduction, as measured by MPE change in the adaptation phase, could be due to compensatory mechanisms specific to the force fields (i.e., real motor adaptation) or by simply stiffening the reaching limb. To eliminate the possibility of such a generic and nondirectional strategy, we compared the difference in MPE between the end of baseline and beginning of washout within and across groups. Participants who adapted to the force fields should have exhibited aftereffects once the force field was removed (e.g., ref. 33). Conversely, if participants had merely adopted a stiffening strategy, no deviation from straight reaching should be observed when no force field is present. Indeed, all paired *t* tests within groups were significant (*t*(19) = 15.21, *P*_active_ = 2.1e-11; *t*(19) = 9.82, *P*_MI_ = 2.8e-08; *t*(19) = 3.97, *P*_control_ = 0.0008), indicating that aftereffects were present, albeit to a different extent. Participants in the active group showed greater aftereffects than participants in the MI (t(18)=−5.73, *P* = 2.7e-06) and control group (*t*(18) = −12.74, *P* = 1.6e-14); and participants in the MI group showed greater aftereffects than participants in the control group (*t*(18) = −7.10, *P* = 5.3e-08; see Fig. 2*D*). Contrary to the comparison of performance at the beginning vs. end of adaptation, this result suggests that even control participants were able to benefit from the visual cue to counteract the interfering force fields to a small extent. Imagining prior movements, however, allowed a much stronger adaptation, and performing overt prior movements resulted in the strongest effects.

Another measure that is often reported in adaptation experiments refers to the predictive compensation participants exhibit after experiencing force fields (e.g., ref. 28). In our reaching tasks, two trials of each block were clamp trials, in which the exoskeleton robot restricted reaches to a straight line from the middle to the final target. We measured the force the robot needed to apply to keep participants on the straight trajectory. If a participant had learned to counteract a force field, then the force the exoskeleton robot must apply in clamp trials should be of equal magnitude. Accordingly, a value of 100% force field compensation (FFC) would indicate that a participant learned to perfectly counteract the previously experienced force fields. We observed the same adaptation pattern of averaged FFC per group over blocks as with the MPE (Fig. 2 *E* and *F* and *SI Appendix*). Taken together, imagining a prior movement, associated with force field direction, allowed motor adaption, albeit less robust than explicitly making the prior movement. The same perceptual information, without a prior movement (imagined or executed) led to minimal adaption. Motor imagery is thus an effective contextual cue for force field-specific learning and can substitute for an active prior movement to some extent.

### Timing Check Task.

Next, we asked whether a hybrid practiced sequence would enhance performance of the whole overt sequence production. To approach this question, all participants performed two active reaches in a timing check task. Instructions were identical to those for the active group in the main reaching task (*Materials and Methods*). We compared, between groups, the median reaction times and dwell times in the middle target. A permutation test was used due to skewed data.

All groups initiated their reaches to the middle target similarly fast (all group comparisons for reaction time medians *P* > 0.16; *Md*_active_ = 379.75 ms; *Md*_MI_ = 396.25 ms; *Md*_control_ = 373.25 ms). However, we reasoned that faster transitions between the segments should be apparent after a sequence is learned (34). Accordingly, we analyzed how long participants dwelled at the middle target in the timing check task (Fig. 2*G*). Participants in the active group spent less time at the middle target than the MI (*P* = 0.0321) and control groups (*P* = 5.2e-05). This is not surprising, given that the timing check task was identical to the main reaching task for active participants and, thus, they had ample training. Critically, however, the MI group also dwelled for a shorter time at the middle target than the control group (*P* = 0.0403). Even though perceptual information had been identical for the two groups in the main reaching task, MI participants were faster to connect the two reaches in the timing check task. This indicates that participants in the MI group used the hybrid sequence as training for the full execution of the same sequence, implying that they formed a representation of two linked movements in the reaching task, even though they only imagined the first segment. The imaginary training, thus, allowed the MI group to connect the two overt movements in the timing check task faster than the control group.

In addition, we analyzed to what extent reaches were fused together based on their velocities (see *SI Appendix* for details). Mirroring the dwell time analysis results, the active group showed the most fusion between reaches and crucially the MI group also showed more fusion than the control group (*SI Appendix*, Fig. S1), indicating that the training of hybrid sequences translated to more efficient production of two reaches with less velocity slow-down between reaches. The differences between groups in the dwell time as well as the fusion index are especially remarkable because participants were not instructed to perform reaches as fast after each other as possible but instead to adjust their reaches to the color changes of the targets. Taken together, motor imagery of a prior movement was beneficial for both the adaptation to interfering force fields and movement execution of the whole sequence.

### Neural Oscillatory Changes During Motor Imagery.

Next, our objective was to examine the potential association between neural markers of motor imagery proficiency and adaptation performance. For this, we recorded electroencephalography (EEG) with 60 electrodes and electromyography (EMG) of right arm muscles during the entire experiment. To measure neural activity during motor imagery, without interfering signals from motor planning for an upcoming reach, participants of the MI group additionally performed the imagined fist clenching task (Fig. 1*D*). At the start of this task, a white fixation cross was displayed. After 2 s, the cross turned red, signaling participants to imagine tightly gripping the handle with their right hand for 3 s, until the cross turned white again (Fig. 1*F*). This sequence of events was repeated 40 times. 16 participants of the motor imagery group performed this task successfully (see *Materials and Methods* for details). For this stand-alone task, we decided on imagined fist clenching rather than imagined reaching for several reasons. First, neural correlates of imagined fist clenching are well researched (35–38) and a robust signal-to-noise ratio could be expected. Second, fist clenching of the robot handle is part of the necessary motor output needed for successfully reaching in our experiment. Most importantly, to effectively and robustly capture oscillatory effects that take time to develop, we aimed for a motor task where imagery could be easily maintained for 3 s without adding unnecessary complexity, such as combining multiple reaches.

**Fig. 3.**
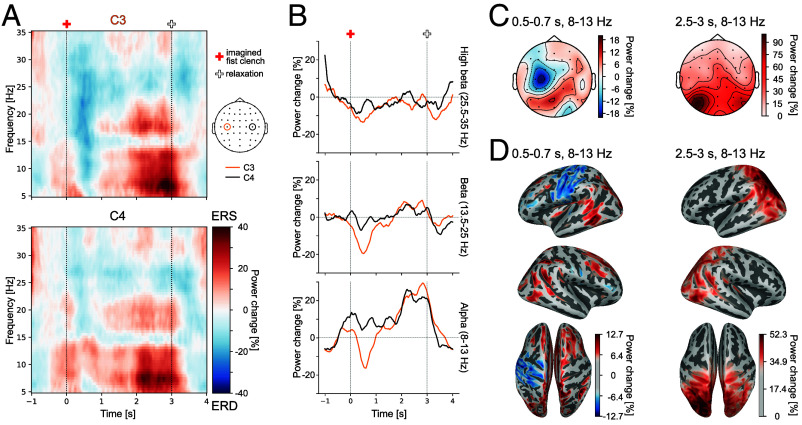
EEG data imagined fist clenching task. (*A*) Time-frequency representation of grand averaged (N = 16) data at electrode standard positions C3 and C4. Color represents power change in percent relative to the baseline window from −0.75 to −0.25 s. Red cross—start of motor imagery, white cross—start of relaxation. (*B*) Power change in percent over time relative to baseline activity in 3 frequency ranges averaged across participants and respective frequencies. Orange line—C3 electrode; black line—C4 electrode. (*C*) Distribution of averaged alpha activity across the scalp in specified time windows (*Left*—ERD; *Right*—ERS), averaged across participants. (*D*) Source reconstruction of averaged alpha activity in chosen time windows, averaged across participants. The strongest 50% of values are shown in color.

We focused on changes in the power of oscillations in alpha and beta frequency bands, which were previously shown to be modulated by motor imagery (5, 39, 40). Therefore, we analyzed the power change during motor imagery starting at 0 s relative to the baseline activity, which was calculated in the interval −0.75 s to −0.25 s. In line with previous studies, we observed a lateralized event-related desynchronization (ERD), in alpha and beta bands, in electrodes over the contralateral sensorimotor cortex after the start of the mental imagery averaged over participants in the imagined fist clenching task (Fig. 3 and *SI Appendix*, Fig. S2).

In the more complex reaching task, we did not observe this typical lateralized motor imagery pattern (*SI Appendix*, Fig. S3), likely reflecting an overlap of neural activity resulting from imagined, planned, and executed movements. Comparing the power changes of the control, MI and active group, differences were, as expected, most evident during the time window in which the task differed between groups (wait, imagine prior movement, or execute prior movement) and after the reaches were completed and feedback was given (*SI Appendix*).

### EEG of Imagined Fist Clenching Task Predicts Motor Adaptation.

To assess how neural data in the imagined fist clenching task related to adaptation performance in the reaching task, we first linearly combined our 3 behavioral adaptation measures into an overall measure of error reduction to maximize robustness and reliability. This included the change of MPE from the beginning to end of adaptation, the change of MPE between baseline and washout, and the FFC at the end of adaptation. Next, we tested the correlation of this change of error with each time-frequency bin in all channels across participants and performed a cluster-based permutation test (initial grouping threshold = 0.01, 1,000 permutations) to investigate the relationship between oscillatory brain activity during the imagined fist clenching task and adaptation performance.

**Fig. 4.**
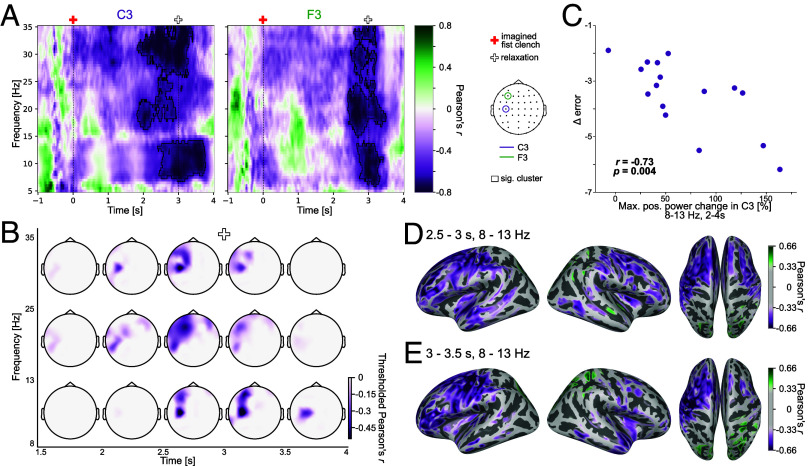
Correlation analysis results. (*A*) Pearson’s correlation of change of error in the reaching task with power change in the imagined fist clenching task across participants in C3 and F3. The pixel color indicates the respective correlation value at that location. A more negative change of error denotes better adaptation performance. Red cross—start of motor imagery, white cross—start of relaxation. The significant cluster is outlined in black. (*B*) Topoplots of different time windows and frequency ranges displaying the distribution of thresholded correlation values. Only the significant cluster is shown. Correlation values are averaged in the specified time and frequency ranges. (*C*) Relationship between change of error in the reaching task and the maximal positive power change value (ERS) in the imagined fist clenching task across participants. Each point depicts a participant. The maximal positive power change value was taken for each participant in the alpha frequency range between 2 and 4 s. (*D* and *E*) Pearson’s correlation of change of error in the reaching task with power change in the imagined fist clenching task—averaged over specified time and frequency range—across participants in source space. The strongest 50% of values are shown in color in (*D*) and the color bar is kept identical for (*E*).

Change of error was negatively correlated with power change (*P*_cluster_ = 0.023). This correlation was driven by power changes from approximately 2 to 3.7 s, which, thus, included a time interval after the termination of motor imagery at 3 s. The cluster comprised both alpha and beta frequency ranges and was most prominent in channels C3 and F3 (see Fig. 4 *A* and *B*; for topoplots displaying unthresholded data see *SI Appendix*, Fig. S4*A*). The single strongest correlations of all single time-frequency bins in C3 and F3 were *r*(14) = −0.891 (at 34.5 Hz, 3.15 s) and *r*(14) = −0.84 (at 8.5 Hz, 3 s), respectively. Negative correlations indicate that bigger oscillatory power increases were associated with more negative changes of error (i.e., stronger performance improvement) in the respective time-frequency-channel bins belonging to the significant cluster. Coaligning the averaged time-frequency representation (for example, C3; see Fig. 3*A*) with the significant cluster (Fig. 4*A*) indicated that a stronger event-related synchronization (ERS) was related to a bigger performance improvement observed during adaptation. We observed similar correlation patterns (lateralized and strong correlations at C3) when we assessed the relationship between the neural data and individual behavioral measures (*SI Appendix*, Fig. S5).

Because different participants may exhibit their peak ERD and ERS responses at different times in our analyzed time window, we tested whether individual alpha peak ERD or ERS values in channel C3 correlated with error reduction across participants. We chose the alpha frequency range for our main analysis because here we observed the largest power modulation (see Fig. 3*C*; for beta see *SI Appendix*, Table S1). The peak ERD did not correlate with the change of error across participants (*r*(14) = −0.116, *P* = 0.67). The peak ERS, however, strongly correlated with change of error (*r*(14) = −0.73, *P* = 0.004; see Fig. 3*C*). Similarly, the overall measure of oscillatory responses based on the peak-to-peak difference of ERD and ERS in each participant correlated with change of error (*r*(14) = −0.722, *P* = 0.003). Thus, stronger ERS in the imagined fist clenching task and a stronger overall modulation of the alpha band power were both predictive of motor adaptation performance in the reaching task. Because the sample size for the correlation analyses was relatively small, we undertook additional analyses to demonstrate that our findings are robust over different analysis pipelines and not dependent on single participants (*SI Appendix*, Table S1).

### Source Reconstruction.

To more precisely identify brain sources, we visualized the correlations of averaged power values in specified time/frequency windows with change of error in source space. In the ERS time window (2.5 to 3 s) the strongest negative correlations were mainly found in the left postcentral and superior-frontal regions (Fig. 4*D*). In the time window right after completion of the imagined fist clenching (3 to 3.5 s) the strongest negative correlations were observed in the left post- and precentral regions (see Fig. 4*E* and *SI Appendix*, Fig. S4 *B*–*D* for correlation’s strength over additional time/frequency ranges).

### No Evidence That EEG of Reaching Task or Subjective Rating of Motor Imagery Predicts Motor Adaptation.

In a next step, we searched for a relationship between oscillatory activity during the imagined movement in the reaching task and motor adaptation performance. However, we did not find a significant correlation across participants when performing the same analysis steps as we did with the imagined fist clenching EEG data (*SI Appendix*). This is likely due to motor imagery and motor planning/execution processes overlapping in the reaching task and therefore obscuring the relationship between oscillatory dynamics and adaptation performance. Even though we argue for a “chunked” representation of the whole hybrid sequence, motor planning of future movements might still occur simultaneously to (imagined) movement execution (27, 41). In the fist clenching task, the ERS at the end of motor imagery was related to adaptation performance. In the reaching task, however, ERS after imagery was absent, potentially being masked by the ERD of the overt following reach. We also found no relationship between EEG power and adaptation performance when incorporating participants of the active group into a linear model that predicts adaptation performance from power and group (main effect power: *P*_cluster_ = 0.59; interaction effect power ∗ group *P*_cluster_ = 0.58; see *SI Appendix*). In addition, we did not find a relationship between perceived vividness of the imagined reach and motor performance or neural data (*SI Appendix*). This suggests that motor imagery proficiency might not be reliably accessible by subjective ratings.

## Discussion

Previous work has already shown that imaginary movements that occurred after (rather than, here, before) the force field-affected reach improved adaptation (42). However, when an imagined segment is instructed to occur last, participants may have planned the full sequence only then to abort its execution just before the final, imagery element. Consequently, they may have associated a full-sequence motor plan with the expected force field (43). Our paradigm excluded this alternative strategy, thereby requiring genuine motor imagery generation. Our findings, including improved adaptation and reduced dwell-time between segments, demonstrate that motor imagery can have benefits beyond just improving target movement performance itself. By incorporating motor imagery in a partly executed motor sequence, training of this hybrid sequence can, on the one hand, facilitate selection of the desired motor response. On the other hand, linking of well-trained hybrid sequences can also transfer to overt sequence production.

Our results thus support the functional equivalence model, which postulates that motor imagery involves generating a complete motor plan that is merely inhibited from use at the execution stage (30). According to this model, neural representations of imagined and executed movements largely overlap, offering a potential mechanism for how imagining one sequence segment can affect performance and learning of linked elements. Here, the motor representation of a well-trained hybrid sequence provided the information required to counteract the forces that arise from dynamics that would be experienced later in the sequence. This is in line with motor imagery activating forward models to predict the associated, hypothetical sensory outcomes (44).

Typically, researchers distinguish between visual and kinesthetic motor imagery (45). Visual motor imagery requires creating a mental image of the body moving, which can be from a first-person perspective (internal imagery, egocentric) or a third-person perspective (external imagery, allocentric). Kinesthetic motor imagery, on the other hand, involves the sensation of how it feels to perform the action, including the muscle movements, joint actions, and the load or force involved (6). In this study, we chose to instruct participants to use kinesthetic motor imagery. This decision was motivated by its resemblance to motor execution in terms of connectivity patterns (46), and the reported modulation of corticospinal excitability, a phenomenon not seen with visual motor imagery (47). Thus, we employed the motor imagery condition that most closely resembles the neural activation of actual movement performance.

Beyond the evident difference in the physical execution and sensory feedback of the movement, overt and imagined movements are clearly distinct. For example, increased activity in one brain area during motor imagery does not necessarily imply increased activity during movement, or vice versa (4, 48). Neural activation during motor imagery also differs depending on the practiced modality (18). In addition, behavioral differences after motor imagery practice and motor execution have been reported before (49, 50). We observed lower adaptation performance for participants performing motor imagery compared to those actively performing the whole reaching sequence. We suggest this is the case because of the weaker neural activation during motor imagery as compared to overt movement (*SI Appendix*, Fig. S3; 5). Weaker activation is likely to have a less pronounced effect on the following movement execution and therefore on learning of the hybrid sequence. A recent ultrahigh-resolution 7T study provided compelling evidence that motor imagery evokes responses in the superficial layers of the primary motor cortex (M1), whereas overt movement evokes responses in both superficial and deeper layers (51). This finding is in line with current concepts of layer-specific organization of M1. Superficial layers receive somatosensory and premotor input, whereas cortico-spinal output, needed for actual movement, is primarily derived from deep layers (52). Interestingly, in rats, the activation of deep layers in M1 seems to be critical for successful motor learning (53). Speculatively, then, the difference in adaptation performance we observed between participants performing an overt as compared to an imagined prior movement may result from varying activation patterns in the neural layers of M1, consistent with the strong, localized effect of imagery over the sensorimotor cortex in our EEG data. Whereas engagement of only superficial layers of human M1 may principally be sufficient for motor adaptation to occur (as in the motor imagery group), evoking responses from deep layers (when performing an overt movement) may further support adaptation.

Alpha and beta ERD are presumed to reflect cortical excitability and are considered biomarkers for cortical and spinal engagement of the motor system (54), and accordingly, improved motor performance has typically been observed to coincide with enhanced ERD (55, 56). The same is true for motor imagery, with ERD patterns of actual and imagined movement being highly similar, suggesting that their neural underpinnings are shared (5, 21, 23). Moreover, subjects have given higher subjective imagery ratings the more similar their ERD in imagined and executed movements (40). In contrast, ERS, rather than ERD, has been observed at the end of imagined movements, particularly in the beta range (57). This signal may indicate a resetting of the sensorimotor system, with higher ERS reflecting enhanced neural processing efficiency, or disengagement from the imagined action (58). Thus, ERD may reflect movement simulation, whereas ERS may rather reflect efficient temporal control and the ability to terminate the imagined action (see ref. 59).

Correlations between the power of oscillations in the imagined fist clenching task and motor adaptation were spatially widespread. A network involving contralateral sensorimotor and prefrontal regions showed strong correlations in both the alpha and beta bands. The ventral premotor cortex (PMv) plays a crucial role in encoding actions, particularly those related to hand movements and object manipulation (60, 61). Motor imagery studies have consistently shown activation in the PMv (4, 62). This activation might be specific to storing movement concepts or reflect other processes such as motor preparation, simulation, or motor awareness to maintain the boundary between motor imagery and actual movement execution (63, 64). Activity in the dorsolateral prefrontal cortex (DLPFC) has also been consistently reported during motor imagery (4) and is likely related to higher-level control processes. DLPFC activity (65), as well as M1 (66), has been related to movement inhibition, fitting with the functional equivalence model’s requirement to forego execution of planned movements. Others have attributed DLPFC activity to adaptive cognitive control (67), in line with the motor-cognitive model of motor imagery, which suggests that imagining a movement uses more executive resources than actually performing the movement, due to the lack of sensory feedback (68). Moreover, the motor simulation theory postulates that higher cognitive systems interact with and supervise the motor simulation process (31). The lack of sensory feedback may necessitate the engagement of higher-level control processes during imagined execution. The ERS we observed in the fist clenching task may, thus, reflect sufficient engagement of executive control during imagery to simulate the movement. As motor adaptation also relies on executive functions (69), superior executive function may be associated with both stronger ERS in the imagined fist clenching task and better adaptation performance in the reaching task. Finally, successful motor adaptation requires the somatosensory cortex (70). Individual trial analysis of a joystick motor adaptation task revealed that the magnitude of the postmovement ERS in beta band correlated negatively with the degree of error during the movement (71). The ERS has been interpreted as an indicator of confidence in internal feedforward estimation during Bayesian sensorimotor integration (72). Thus, the ability to form and update motor memories might be reflected by both the adaptation performance in the reaching task and by synchronization of oscillatory activity in the somatosensory cortex after an imagined fist clench, contributing to the observed correlation pattern.

Different motor imagery training tasks, such as imagined finger sequencing (73), isometric force production (74), goal-directed reaching (21), gait (75) and muscle strength (9) have been employed to explore performance enhancement through imagery interventions and/or underlying neural mechanisms during the same target task. Our findings, including improved adaptation and reduced dwell-time between segments, demonstrate that motor imagery can have additional benefits beyond just benefiting target movement performance itself. By incorporating motor imagery in a partly executed motor sequence, training of this hybrid sequence can, on the one hand, facilitate selection of the desired motor response. On the other hand, linking of well-trained hybrid sequences can also transfer to overt sequence production.

Our results open exciting possibilities for sports training and rehabilitation. For example, in stroke recovery, gross motor skills, such as arm reaches, often recover before fine motor skills, such as finger movements (76). In this context, training a hybrid sequence consisting of, for example, an overt reach to an object and an imagined grasping movement (or vice versa), may facilitate the relearning of the grasping movement through the well-researched effects of motor imagery of the target grasping movement (e.g., by stimulating the same neural pathways as actual movement and promoting brain plasticity). Moreover, with practice, the prior reaching movement can cue the specific (imagined) grasping movement and thereby further facilitate the learning process. Finally, the transition between reaching and grasping might be improved if both movements are overtly performed in sequence after hybrid training.

It will be crucial to assess which types of movements are suited for applied hybrid sequence interventions and to determine what the best training approaches are. In the given example, patients might benefit more if they imagine (or execute) a specific prior reach trajectory linked to a specific (imagined) object grasp, or, alternatively, if they practice various sequences. Future research should also identify individual differences and prerequisites for effective interventions. Functional imaging studies will be needed to delineate more precisely the role of different brain areas in motor imagery, adaptation, and sequence production processes and their contribution to learning of hybrid sequences. In our study, subjective vividness of the imagined movement did not relate to performance improvement or to a neural correlate. This suggests that self-report might not sufficiently capture the aspects of motor imagery that relate to performance (42, 77).

To summarize, our study demonstrates that hybrid sequences combining overt and imagined movements can significantly enhance adaptation to interfering force field perturbations. Accordingly, motor imagery can be leveraged to improve the performance of not only the imagined but also of linked overt movements. In addition, we have established a clear connection between motor adaptation ability and neural oscillatory dynamics observed during an unrelated motor imagery task. Notably, individuals who exhibited stronger oscillatory modulation during a basic motor imagery task showed greater adaptation improvement in overt movements that were linked to prior imagined movements. This finding underscores the importance of individual differences in motor imagery proficiency, suggesting that the effectiveness of imagery-based motor adaptation may be influenced by these neural dynamics.

## Materials and Methods

### Participants.

A total of 65 volunteers aged 18 to 35 y participated in our study. We excluded 5 participants from our analysis, as explained below. Therefore, our final sample consisted of 60 participants (30 females, 30 males) with a mean age of 26.1 (SD = 4.6) years. All participants were right-handed and had normal or corrected-to-normal vision and no known neurological, perceptual, or motor impairments or disorders. The study received ethical approval from the local ethics committee at the University of Leipzig, and all participants provided informed written consent prior to the experiment.

### Apparatus and Stimuli.

The tasks were performed within a Kinarm Exoskeleton Lab (Kinarm, Kingston, Canada). This robotic device can reliably track arm movements in the horizontal 2D plane with a recording rate of 1,000 Hz. Targets and individually calibrated hand positions are displayed via a mirror reflecting a monitor mounted above (Fig. 1*A*).

We measured EEG with 60 passive electrodes (Brain Vision by Brain Products, Gilching, Germany) following the international 10 to 20 system. In addition, we measured electrooculogram (EOG) with 4 electrodes, electrocardiogram (ECG) with 2 bipolar electrodes, and EMG of the right arm and shoulder muscles (brachioradialis, triceps lateral head, pectoralis major, posterior deltoid) with 8 bipolar electrodes. The sampling rate for all recorded biological signals was 2,500 Hz. All electrodes were attached to the participants before they entered the Kinarm robot.

### Tasks and Procedure.

Participants were randomly assigned to the control, active, or MI group (Fig. 1*B*). Each participant performed a reaching task that was slightly different depending on the group. The subsequent timing check task was the same for everyone. Participants in the MI group additionally performed the imagined fist clenching task (Fig. 1 *D* and *F*). Last, participants were asked to fill out a short questionnaire.

#### Reaching task.

Participants performed right-arm reaches toward targets in the Kinarm robot. Participants were given a brief introduction to the task before starting the experiment. They then completed a total of 60 blocks, consisting of 6 baseline, 50 adaptation, and 4 washout blocks (Fig. 1*D*). Each block included 16 normal and 2 clamp trials. During normal trials, in the adaptation phase, force fields perturbed the reaching movement between the middle and final targets. The robot’s perturbations in the adaptation phase appeared random to the participants included in this study.

In total, participants performed a minimum of 1,080 trials. Short breaks (1 to 3 min) were taken approximately every 200 trials, and there was also a 5-min break at the halfway point. Depending on the individual participants, the task took between 1, 5, and 2 h. Example videos of the reaching task of each group can be found on OSF (https://osf.io/swgd9).

During each reaching trial, three targets were presented: the cue, middle, and final target (for size/position details, see *SI Appendix*). Depending on the group, a trial consisted of either one or two active reaches. Participants in the active group performed two successive overt reaches. The timing was indicated by color changes of the different targets. Participants in the MI group were asked to imagine the first reach in each trial. More specifically, they were instructed to perform kinesthetic motor imagery from the first-person perspective, in other words to try to mentally feel the muscle movements and sensations associated with performing the reach, without actually moving. Immediately after the imagery effort, they performed an active final reach. Participants in the control group performed only one reach. The only difference between the MI and control group was the instruction to perform motor imagery for the former group. In every trial, all experimental groups performed the same final reach, which involved moving the right hand from the middle to the final target. A velocity-dependent curl field was sometimes present between the middle and final target causing systematic perturbations to the arm during those reaches. The force field began after the right hand was more than 2 cm away from the middle target’s midpoint and remained present until the final target was reached. The location of the cue, relative to the final target, determined the direction of the force field. To account for any kinematic or biomechanic advantages, half of the participants learned that a positive angle between the cue and final target corresponded to a clockwise (CW) force field, while the other half learned that it corresponded to a counterclockwise (CCW) force field. This association between the angle’s sign and the direction of the force field remained constant for each participant throughout the experiment. The experienced forces *F* were perpendicular to movement direction and varied based on reaching velocity:

$$\left[\begin{array}{c} F_{x}\\ F_{y} \end{array}\right]=c\left[\begin{array}{cc} 0 & -1\\ 1 & 0 \end{array}\right]\left[\begin{array}{c} \dot{x}\\ \dot{y} \end{array}\right]$$

Depending on the location of the cue, the constant c was set to −13 or 13 Ns/m (78). There was never a force field between the cue and the middle target.

In randomly selected trials of the reaching task, the Kinarm robot enforced straight movements between the middle and left final target by using a force channel. We refer to these trials as clamp trials because the force channel walls restricted participants’ movements to a straight path. If participants adapted to the previously experienced force fields, they would anticipate the direction and strength of the fields and counteract the perturbations. In clamp trials, the Kinarm robot measured any compensatory forces applied by the participants against the channel walls. This allowed us to assess any potential feedforward learning (for complications see *SI Appendix*).

Each group consisted of 20 participants (10 females, 10 males; for more information + detailed trial sequence description see *SI Appendix*).

#### Timing check task.

In the timing check task, all participants performed two reaches: from the cue to the middle and from the middle to the final target. There were two blocks with 24 trials each (for more information, see *SI Appendix*).

#### Imagined fist clenching task.

The imagined fist clenching task at the end of the experiment was only performed by participants in the MI group. Participants were still seated in the Kinarm robot. At the start of the task, the robot moved participants’ right hand to the former position of the middle target, where now a white fixation cross was displayed. After 2 s the cross changed color from white to red and participants were instructed to imagine clenching their fist around the handle as hard as they could, as long as they saw the red cross. After 3 s, the cross changed back to white. The participants were instructed to remain in a relaxed state during the presentation of the white cross. This sequence was repeated 40 times.

We introduced the imagined fist clenching task after a break in data collection due to COVID-19 pandemic restrictions. For this reason, the first three MI participants did not perform the task. Another participant had to be excluded because they contracted their muscles during the 3 motor imagery seconds (captured by EMG; see *SI Appendix*). The sample of the imagined fist clenching task consisted therefore of 16 participants.

#### Questionnaire.

At the end of the experiment, participants filled out a questionnaire. One objective was to find out whether participants had noticed any specific pattern between the force field direction and the cue position and whether they were explicitly aware of it. As a result, five participants (three from the MI group, two from the control group), who correctly identified this association, were excluded from the analysis and additional data from five new participants were acquired to replace them.

A second objective was to obtain a subjective rating from participants in the motor imagery group about their perceived ease/difficulty of imagining the movement in the reaching task. For this, we adapted the questions from the widely utilized motor imagery questionnaire (MIQ-RS, 79) to our task. Specifically, participants rated how well they thought they imagined the movement on average over the whole reaching task on a 7-point Likert Scale. The scale (in German) ranged from 1 (“extremely difficult to feel”) to 7 (“extremely easy to feel”) with a neutral option at 4 (“not difficult/not easy to feel (neutral)”).

#### Data analysis.

For the detailed data analysis see *SI Appendix*, *Extended Methods*.

### Preregistration.

We preregistered our study design of the reaching task, which includes sample sizes, general hypotheses, and the main kinematic analysis plan, on OSF (https://osf.io/swgd9). Furthermore, exemplary reaching task videos are available there.

## Supplementary Material

Appendix 01 (PDF)

## Data Availability

Anonymized kinematic data and EEG data of the imagined fist clenching task have been deposited in OSF (https://osf.io/swgd9; 80). In addition, the code used for data processing, analysis, visualization is also available (80).
