# Supplementary material for: Motor imagery enhances performance beyond the imagined action
Source: Proc Natl Acad Sci U S A. 2025 May 13;122(20):e2423642122. doi: 10.1073/pnas.2423642122 (PMC12107166; doi:10.1073/pnas.2423642122)
Supplement: Supplementary file 1 — Appendix 01 (PDF) [file pnas.2423642122.sapp.pdf]

Supporting Information for

Motor Imagery Enhances Performance Beyond the Imagined Action

Magdalena Gippert, Pei-Cheng Shih, Tobias Heed, Ian S Howard,

Mina Jamshidi Idaji, Arno Villringer, Bernhard Sehm and Vadim V Nikulin

Magdalena Gippert
E-mail: gippert@cbs.mpg.de

This PDF file includes:

Extended Methods
Supporting Information Text
Figs. S1 to S5
Table S1
SI References

Extended Methods

Target details. All targets had a diameter of 1.25. The location of the middle target was adjusted based on the individual's hand position when the elbow was bent at a 90° angle and the shoulder angle was at 60°. There were four potential positions for the final target, located either 12 cm to the right, left, up, or down from the middle target. For each final target position, there were two possible cue positions (see Fig 1E). The distance between the cue and the middle target was 10 cm. The current hand position was indicated by a red cursor with a diameter of 0.5 cm. The cue and middle target were gray and changed to white during the trial, and the final target was yellow. The targets' number, color, size, timing, and between-target angles were determined based on previous research (e.g., 1 and the same as in 2).

Clamp trial complications. Due to technical limitations, the force channel walls were sometimes not strong enough. As a result, participants were able to slightly deviate from the straight line, and in some cases, they even broke through the virtual wall and moved off the intended trajectory. These trials were excluded from our analysis. Performance of the last four adaptation blocks was, thus, based on 407 instead of 480 total trials.

Trial sequence. The motor imagery group had a mean age of 25.75 ($SD = 4.46$) years. During each trial in the motor imagery group, a white fixation cross appeared on a black background, and the robot moved the participants' right arm towards the middle target (see Fig. 1C). This passive positioning lasted for 1000 ms. Following this, all targets (cue, middle, and final) and the hand position cursor were displayed. After a random period of time ranging from 1000 to 1400 ms, the color of the cue changed from gray to white. This was the signal for the motor imagery group to start imagining moving their hand from the cue position to the middle target. Participants were instructed to imagine the movement, or more specifically, to try to "feel" the movement and how this feeling differed depending on the cue's location. To make this reaching imagery easier, participants executed the actual reach from the cue to the middle target in the practice trials, before the start of the reaching task. 600 ms after the cue color change, the color of the middle target changed from gray to white, indicating that participants should have concluded their imaginary reach and should start to actually move to the final target. Once they reached the final target, feedback on their movement speed was displayed just above the middle target. If the time to move from 2 cm away from the middle to the final target fell between 150 and 300 ms, the feedback (in German) was "good". Otherwise, if it was faster or slower than this range, the feedback was "too fast" or "too slow" accordingly. The feedback was shown for 600 ms. Subsequently, a white fixation cross was displayed for 400 ms before the next passive positioning for the next trial began. When half of the trials in a block were completed, the inter-trial interval was extended to 4 s instead of 400 ms. If the cursor was not in the middle target when the color changes of the targets occurred, the trial was immediately aborted and repeated within the same block. Additionally, if participants left the middle target earlier than 100 ms before, or later than 500 ms after, the *go*-signal, the trial was considered unsuccessful and was repeated at a random position within the current block.

For two participants in the motor imagery group, the time between cue color change and middle target color change was 400 ms instead of 600 ms. However, their performance was comparable to the other participants in the group, with all three dependent variables (explained below) deviating by less than 1.4 standard deviations from the mean of the 600 ms group. Therefore, their data was included in the kinematic but not in the EEG analysis.

The timing, feedback, and repetition criteria were consistent across all groups. The control group was presented with the same trial procedure but was not told to do anything until the middle target changed color. In the active group, the participants' hands were moved to the cue's position at the start of the trial and they were instructed to make two subsequent reaches: from the cue to the middle target and from the middle target to the final target. The control and active groups have been previously described in greater detail in (2). In addition to the task, participants performed 5 min of open eyes resting state before, in the middle and after the reaching task.

Additional details timing check task. The timing of color changes and abortion criteria were the same as in the reaching task. However, there were no clamp trials and no force fields. Another slight deviation was the position of the cue. In 33% of trials, the cue was moved to 75% of the original 10 cm distance from the cue to the middle target (7.5 cm) and in another 33% of trials, the cue was moved further away (13.3 cm), so that the original 10 cm distance equaled 75% of the longer distance. This is was done to introduce more variability and therefore difficulty in the task. Every unique cue/final target combination was presented three times, once for each tested distance between the cue and the middle target.

Due to the individual set-up and calibration, nine participants could not reach the cue's position in the upper-left corner when it was moved further away (13.3cm) from the middle target. The non-executable trials were excluded and a block consisted of 22 trials for these participants. Two participants expressed the wish during the experiment to finish early and thus only executed one block instead of two.

Magdalena Gippert, Pei-Cheng Shih, Tobias Heed, Ian S Howard,
Mina Jamshidi Idaji, Arno Villringer, Bernhard Sehm and Vadim V Nikulin

Data analysis.

Kinematic analysis. ***Preprocessing.*** The Kinarm device recorded angles of the elbow and shoulder joints at all time points (1000 Hz). We preprocessed the data in MATLAB (R2021a). A low-pass filter was applied to the data using a cutoff frequency of 10 Hz. We added hand velocity, acceleration, and commanded forces to the automatically recorded hand position. Commanded forces refer to the forces generated by the Kinarm device to ensure the participants' hand remained within force channels during clamp trials.

Our main analyses were conducted using Python (version 3.10) and relied on various libraries, including NumPy (3), Pandas (4), SciPy (5), scikit-learn (6), as well as Matplotlib (7) and Seaborn (8) for data visualization. We excluded all aborted trials in our analysis.

Maximal perpendicular error (MPE). One of the primary measures of interest was the Maximal Perpendicular Error (MPE), which quantified the maximal deviation in cm from a straight line connecting the middle and final targets of the right arm trajectory. A positive value indicated that participants exhibited a curved trajectory in the direction of the force field. The MPE was calculated from 2 cm past the middle target's midpoint (toward the final target) and the final target's endpoint. We excluded trials in relevant blocks in which it was evident that participants had chosen an incorrect target at the start (9 trials across all samples).

To assess how well each participant adapted to the force fields, we investigated two variables related to the MPE. First, we subtracted the average MPE of the first adaptation block from the average of the last two adaptation blocks (MPE change adaptation, see Fig. 2C). If the MPE change adaptation value was negative, it meant that the participant's reaches became straighter (less perturbed by the force field) at the end of the adaptation phase compared to the beginning. A more negative value indicated a greater improvement in performance. Second, we calculated the difference between the average MPE of the last baseline and the first washout block (MPE change baseline/washout, see Fig. 2D). If this value was negative, it meant that the participant made consistently more curved reaches in the washout block in the opposite direction of the force field experienced before, compared to the baseline block. This would have been due to a residual counteracting effort that was applied earlier. Consequently, a more negative value indicated a larger force field after-effect.

We performed t-tests between groups and within groups, against zero, for both MPE change adaptation and MPE change baseline/washout. We applied the Bonferroni-Holm correction to adjust the p-values of each family of tests involving the same dependent variable (6 tests each).

Force field compensation (FFC). A second outcome measure was force field compensation (FFC) in clamp trials. To determine FFC, we analyzed force data within a 150 ms time window centered on the time of peak velocity during the reach to the final target. Based on the movement's velocity, we calculated the ideal force profile, which would have counteracted a present force field. We linearly regressed the measured force against the channel walls on the ideal force profile with the intercept forced to zero. We then defined FFC as the slope of the regression multiplied by 100%.

Next, we determined the average FFC for each participant in the final four blocks of the adaptation phase (referred to as FFC final adaptation, see fig. 2F). A final adaptation value of 100%, would mean that the participant made perfect adjustments to their reaches to counteract the force fields. We used FFC final adaptation to again perform t-tests within and between groups and adjusted with the Bonferroni-Holm correction (6 tests).

Timing check task.

In the timing check task we defined reaction time as the time between the *go*-signal, indicated by the color switch of the cue, and the time when participants left the cue location. Dwell time, on the other hand, was defined as the time between entering and leaving the middle target. For each participant, we recorded the median reaction and median dwell time of all performed trials. We compared the two measures between groups with permutation tests. For this we randomly permuted the group labels and calculated the difference of medians one million times to create a null distribution for each comparison. The observed differences between groups were then compared to the respective null distributions to determine the p-values. Note that we did not correct for multiple comparisons here. This analysis was not preregistered and hence exploratory.

EEG analysis imagined fist clenching task. We performed our EEG data analyses using MNE Python (9) and custom scripts.

Preprocessing. The raw EEG data were downsampled to 250Hz (using the *resample* function of MNE Python, which applies a low-pass anti-aliasing FIR filter) and then re-referenced to the average reference. Next, the data were bandpass filtered between 0.5-45 Hz to remove unwanted frequency components using an 8-order Butterworth filter applied forward-backward to prevent phase distortions in data. Subsequently, a notch filter was applied to remove the 50 Hz power line noise with a frequency range of 50 Hz to 125 Hz, filtering the first and second harmonics. Noisy channels were excluded by visual inspection of data for each participant. The maximal number of excluded channels per participant was three.

Independent Component Analysis (ICA) was used to remove noisy components from data. For this, a copy of the data was filtered between 1 and 45 Hz to remove slow-frequency drifts (10). Independent components were estimated with MNE Python using the method *picard* and the fitting parameters set to obtain extended Infomax solutions. The algorithm included a principal component analysis (PCA) to whiten the data before performing ICA. The number of components was set to the number of available EEG channels minus one. Noisy ICA components were removed by visual inspection. Artifactual components included mostly eye movements and muscle activity. The remaining components were projected back to the sensor space. Finally, the continuous data were then segmented into epochs ranging from 1.5 seconds prior to the onset of motor imagery to 4.5 seconds afterward. Noisy epochs and epochs in which the EMG showed muscular activity in the right arm in the imagery compared to the relaxation phase were removed. One participant had to be removed entirely because they clearly

contracted muscles in each motor imagery section. Finally, excluded bad channels were interpolated using MNE Python with *spline* method.

Time-frequency transformation. We computed the current source density (CSD) to estimate spatially specific neuronal activity. To analyze changes in neural oscillations over time and across various frequency bands, we used MNE Python to compute the time-frequency representation (TFR) of the data. A multitaper approach was applied to calculate the power of neural activity for each frequency bin and time point (11). The frequency range for the TFR was set between 5 and 35 Hz, with a step size of 0.5 Hz. The number of cycles for each frequency bin f was set to $f/2$. Within each participant, we calculated the average power across epochs in each frequency-time bin. To reduce memory usage we used a decimation factor of 2 after time-frequency decomposition. We cropped the resulting TFR data from -1 to 4 s.

We performed a baseline correction by expressing the TFR data as a percentage change relative to the mean baseline activity from -0.75 to -0.25 s in relation to the start of motor imagery. This way, we ensured that the subsequent analyses focused on changes in neural activity relative to a time interval in the relaxation phase prior to the motor imagery onset.

Source reconstruction. To obtain more precise information about the spatial location of neural sources, we mapped our data to source space. A forward solution was calculated based on *fsaverage* standard head model, the three-layer boundary element model (BEM), and the dipole grid with ico5 spacing accompanied by MNE Python (12). The resulting source space consisted of 10242 dipoles per hemisphere. We computed the Minimum Norm Estimate (MNE) (13) inverse solution using MNE Python, with dipole orientations normal to the cortical surface and the regularization parameter equal to 0.05. Next, we performed the same time-frequency analysis on the source estimates in source space as on the sensor data (see time-frequency transformation). We used a frequency range of 8-13Hz for the alpha band, and 14-25Hz for the beta band, with a step size of 1 Hz. We used the same baseline correction approach as in the sensor data analysis.

EEG analysis reaching task. We performed the same preprocessing and time-frequency transformation steps as described in the fist clenching task. The data was segmented into epochs from -1.5 to 3 s relative to the cue color change, i.e., the *go*-signal to imagine the reach to the middle target. After the time-frequency transformation, we cropped the TFR data from -1 to 2.5 s and set the baseline window to -0.75 to -0.25 s in relation to the start of the imagined reach.

Correlation analysis imagined fist clenching task. To relate the neural data of the imagined fist clenching task to performance in the reaching task, we combined MPE change adaptation, MPE change baseline/washout and FFC final adaptation to one behavioral value. Our aim was to obtain one behavioral measure that reliably captured overall motor adaptation performance. For this, we normalized each dependent variable by dividing it by its standard deviation across participants. Next, we added MPE change adaptation, MPE change baseline/washout and the inverse of FFC final adaptation. The resulting behavioral variable was termed change of error. A more negative value denoted a better overall adaptation performance.

We performed a cluster-based permutation test to investigate any possible association between the behavioral change of error and the neural data of the imagined fist clenching task. For this, we first correlated change of error with the calculated TFR value for each time-frequency-channel bin. We set the initial threshold for each correlation to $p = 0.01$. Directly neighboring significant bins in time, frequency, or channel formed a cluster. We summed the t-values of those bins belonging to the same cluster and recorded the biggest absolute value of the sum. To obtain an empirical distribution we permuted change of error randomly across participants and repeated the described steps 1000 times. Finally, we compared the originally observed biggest cluster mass to the empirical distribution. Here, we used the standard p-value cutoff 0.05 (see Fig. 4A & B; note that due to thresholding, correlation values are partially underestimated in 4B).

In addition, to account for different time courses in the neural signature of participants, we calculated the correlation of change of error and the largest positive (ERS) and largest negative (ERD) power change value in channel C3. We picked C3 because it is over the primary motor cortex, contralateral to the imagined movement side, and therefore, is usually investigated in motor imagery research as the sensor most reliably picking up activity from the motor cortex (e.g., 14). Since we observed the greatest power modulation averaged over participants (see Fig. 3C) in alpha band, we took the maximal and minimal power values between 8 and 13 Hz. Also based on the average strength of ERD and ERS, we selected the peak ERD value in a time window between 0 and 2 s and the peak ERS value in a time window between 2 and 4 s. Lastly, we also calculated the correlation of change of error and the difference between peak ERS and ERD. We applied the Bonferroni-Holm correction to adjust the p-values of the 3 correlation tests.

To illustrate the correlation of the strength of ERS with adaptation performance in source space, we first averaged the power change of each participant over the ERS time window (2.5 - 3s) and the alpha frequency band (8 - 13Hz) for all sources. Next, we correlated the average power change of each source with the change of error in the adaptation task across participants (see Fig. 4D). We performed the same calculation steps for the subsequent time window (3 - 3.5s; see Fig. 4E). To observe the correlation strength over different time and frequency ranges in source space, we also plotted the average correlation of 1s segments in the examined frequency ranges (see Fig. S4 B, C, D).

Correlation analysis reaching task. We performed an analogous analysis to relate the neural data of the reaching task to the change of error in the same task. In addition, we performed a cluster-based permutation test relating change of error to neural data and experimental group in a linear regression. We fitted a linear model to predict individual adaptation behavior from relative power, group, and their interaction (change of error $\sim$ EEG data $*$ group). We included active and motor imagery participants, excluding the control group due to a lack of task learning. The model was fit for each time-frequency bin between -1 and 2.5 s and 5 to 35 Hz (the step size of the TFR was 1 Hz). We conducted a Type III ANOVA for each bin to assess

predictor significance, providing F-statistics and p-values, with a significance level of alpha $= 0.01$ for clustering. We evaluated the main effect of power and the interaction of group and power separately by summing the F-statistics of neighboring bins (in time, channel position, and/or frequency) to identify the largest cluster, respectively. We then permuted the dependent variable, change of error, and repeated the analysis to obtain a distribution of F-values to compare our observed F-cluster-statistics with.

Correlation analysis questionnaire. Lastly, we investigated if there was any association between the self-reported ease/difficulty of imagining the feeling of the movement in the reaching task and the degree of motor adaptation. For this, we correlated the subjective imagery rating with the change of error across participants. In addition, we repeated the described cluster-based permutation tests above but replaced the change of error with the subjective imagery rating. Again, we set the initial threshold to 0.01. However, we only permuted the data 100 times to save resources as it was evident that significance would not be reached in these analyses. Finally, we correlated the subjective imagery rating with the peak ERS, peak ERD, and the difference between peak ERS and ERD in both tasks.

Supporting Information Text

Force field compensation comparison across and within groups. Comparing the average FFC value at the end of the adaptation phase against 0 revealed significant within-group effects for the active ($t(19)$ = 27.04, p_{active} = 6.3e-16) and MI group ($t(19)$ = 9,16, p_{MI} = 6.3e-08) but not for the control group ($t(19)$ = 2.22, $p_{control}$ = 0.039; see Fig. 2F). Similarly, t-tests between groups revealed differences between all groups ($t(18)$ = 17.02, $p_{active/control}$ = 1.4e-18; $t(18)$ = 7.64, $p_{active/MI}$ = 1.4e-08; $t(18)$ = 6.16, $p_{MI/control}$ = 6.9e-07). The active group showed the most predictive compensation, followed by the MI group.

Fusion index between reaches in timing check task. Fusion of two reaches refers to the integration of the distinct motor elements into one seamless action (15). A complete fusion is characterized by no deceleration between the two reaches. We calculated a fusion index by relating the minimum velocity between two reaches to the mean peak velocity of the two reaches in the timing check task:

$$\text{fusion index} = 1 - \frac{\left(\frac{v^1_{\max}+v^2_{\max}}{2}\right) - v_{\min}}{\frac{v^1_{\max}+v^2_{\max}}{2}}$$

with $v^1_{\max}$ and $v^2_{\max}$ denoting the peak velocity during the first and second reach, respectively, and $v_{\min}$ the minimum velocity between these two peaks (15). We cut the velocity data once participant reached the final target, which as a consequence then sometimes coincided with the peak recorded velocity of the second reach. In trials with only one velocity peak the fusion index was set to 1. The index ranged from 0 to 1, with 0 indicating a complete stop between reaches and 1 a fully coarticulated movement.

We compared the median fusion index of participants between groups with a permutation test (one million permutations). We observed the same results pattern as in the dwell time analysis. Participants in the active group fused their reaches more than participants in the MI (p = 0.0134) and control groups (p = 2.9e-06). Crucially, the MI group also fused their reaches more than the control group (p = 0.0121; see Fig. S1). In fact, when relating dwell time and fusion index we observed strong correlations across participants in all groups ($r_{\text{control}}(18)$ = -0.883, $r_{\text{MI}}(18)$ = -0.888, $r_{\text{active}}(18)$ = -0.931; all $p <$ 1e-05)).

Power changes during the imagined fist clenching and reaching task. In the imagined fist clenching task, on a descriptive level, the ERD was strongest in the alpha band (8-13 Hz; see Fig. 3B) over the contralateral left hemisphere (see Fig. 3C). We also observed an event-related synchronization (ERS) at the end of the motor imagery phase which, however, showed a weaker lateralization. Source reconstruction of the ERD in the alpha frequency band revealed that the strongest desynchronization, according to the Desikan-Killiany atlas, was observed in the left post- and precentral region, i.e. the primary sensory and motor cortex, respectively (see Fig. 3D, left). The ERS was also present in the contralateral hemisphere and more pronounced in the posterior parts of the cortex. The synchronization was strongest in the left superior-parietal region (see Fig. 3D, right). In beta frequency bands, topoplots of the ERD and ERS and the respective source reconstructions showed a similar pattern (see Fig. S2 A & B).

In the reaching task, all groups showed an ERD over sensorimotor areas in alpha and beta bands during the overt reach to the final target starting after 0.6 s. In the alpha band, the desynchronization was stronger on the ipsilateral, right side. Crucially, the MI group exhibited a stronger contralateral ERD in the beta band during motor imagery compared to the control group who just waited in this time period. The most notable difference between groups, however, was the strength of a contralateral ERS after the reaches were completed, with the active group exhibiting a strong power increase, the control group a very slight late increase and the MI group showing a power increase in between these two outcomes.

Reaching task performance is not related to neural data during the same task. We did not find a relationship between change of error and average power change in the reaching task in a cluster-based permutation test (p = 0.22). Similarly, we also did not find a significant correlation between adaptation performance and peak ERD ($r(16)$ = 0.177, p =0.482), ERS ($r(16)$ = 0.156, p = 0.536) or peak-to-peak difference ($r(16)$ = -0.019, p = 0.94) in the 600 ms motor imagery time window in alpha band across participants. This lack of significant findings remained the same when we related neural activity in the last 100 trials to adaptation performance, and also when we segmented the EEG data relative to actual movement onset and not to the visual cues indicating the start of the (imaginary) movement. Taken together, we could not find a neural marker during motor imagery in the reaching task predicting the overall motor adaptation performance of participants.

Subjective rating of motor imagery is not related to performance or neural data. To investigate if there was a relationship between perceived proficiency in motor imagery and motor performance or neural data, we asked participants in the MI group about their subjective experience at the end of the experiment. We adjusted questions from a widely used motor imagery questionnaire (MIQ-RS, 16) to fit our task. In particular, we asked participants to indicate how successfully they were able to feel the imagined prior movement on a Likert Scale from 1 ("very hard to feel") to 7 ("very easy to feel") on average throughout the reaching task. If self-assessed motor imagery would correctly characterize the actual ability to perform motor imagery, we would expect to see a relationship between this subjective imagery rating and the degree of adaptation in the reaching task. However, there was no correlation between the imagery rating and change of error in the reaching task across participants ($r(18)$ = -0.030, p = 0.91).

We also investigated the relationship between the subjective imagery rating of the reaching task and the neural response in the imagined fist clenching and reaching task, respectively. We adopted the same approach as before when we related neural

data and change of error. We did not find any significant clusters when performing a cluster-based permutation test over the complete window duration and considered frequency ranges in the imagined fist clenching ($p = 0.25$) or reaching task (0.96). We also did not find any relationship between the imagery rating and peak ERS, imagery rating and peak ERD, or imagery rating and peak-to-peak difference of ERS and ERD in the alpha band in the imagined fist clenching (ERS: $r(14) = 0.33$, $p = 0.21$; ERD: $r(14) = 0.35$, $p = 0.18$; ERS - ERD: $r(14) = 0.16$, $p = 0.55$) or in the reaching task (ERS: $r(16) = 0.09$, $p = 0.722$; ERD: $r(16) = 0.279$, $p = 0.262$; ERS - ERD: $r(16) = -0.214$, $p = 0.394$). Taken together, we did not find any evidence that the subjective rating of motor imagery difficulty/ease is related to performance or a neural manifestation.

Dwell time of motor imagery participants in the timing check task is not related to performance or neural data during motor imagery. We did not observe any relationship between dwell time in the timing check task and change of error in the reaching task across motor imagery participants ($r(18) = 0.255$, $p = 0.278$) or between dwell time and subjective rating of motor imagery ($r(18) = 0.298$, $p = 0.202$). We also did not see any significant clusters relating dwell time to neural motor imagery data during the reaching task (initial grouping threshold = 0.01, 100 permutations, $p = 0.46$) or the fist clenching task ($p = 0.43$). Dwell time between two active reaches does not seem to capture motor imagery proficiency in our task. An obvious reason for this could be that the task was not designed for participants to perform reaches as fast as possible after each other and participants might have prioritized fusion of the two reaches to a different extent diluting any potential relationship across participants within the motor imagery group.

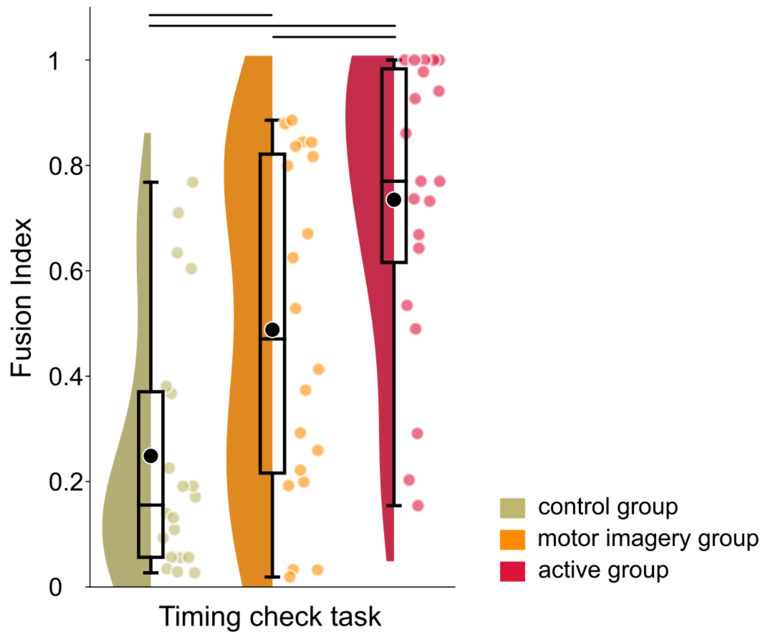

Fig. S1. Comparison of the fusion index in the timing check task. Each colored dot depicts the median fusion index of one participant. Black dots mark the respective group averages. Lines denote significant differences between groups $p < .05$.

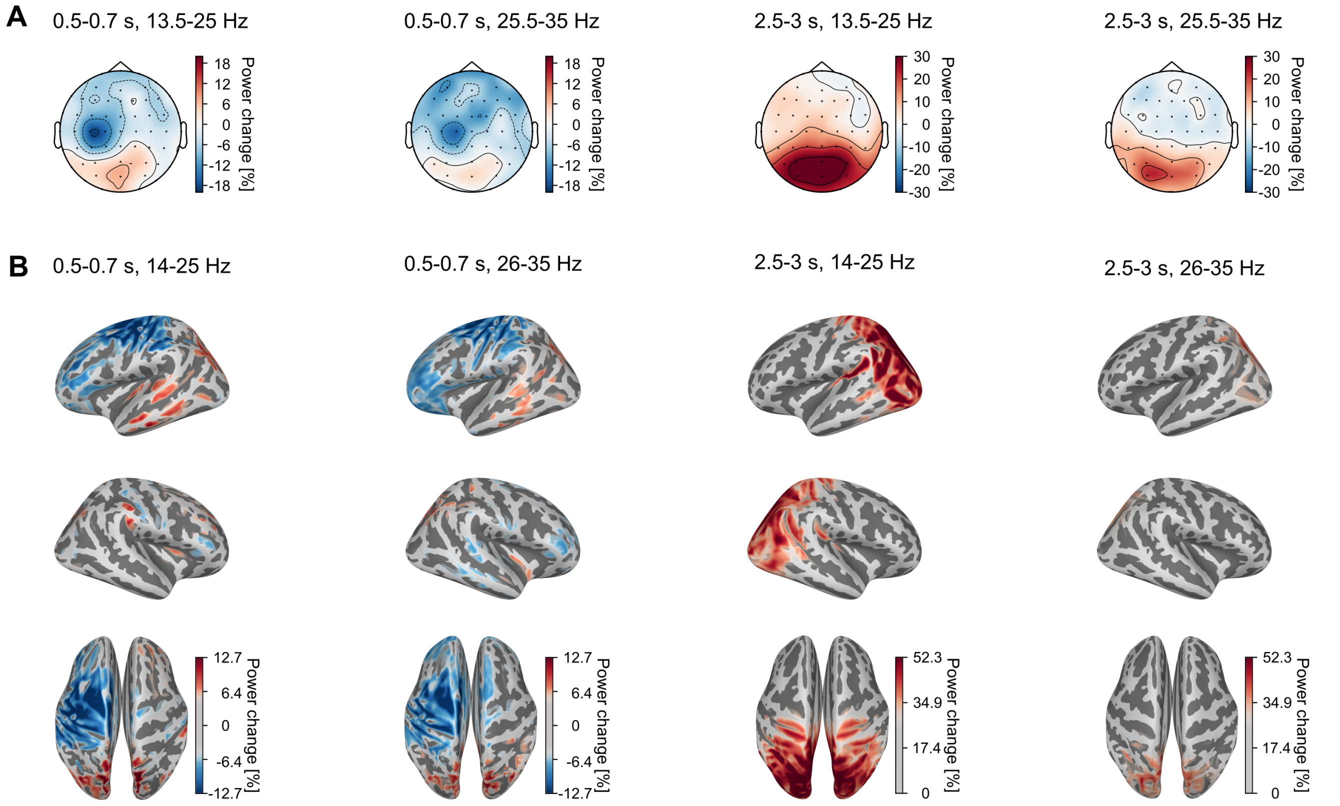

Fig. S2. EEG data imagined fist clenching task (beta frequencies). Wherever possible, the color bars were maintained identical to those in Fig. 3 to facilitate comparison. A) Topoplots of averaged beta and high beta activity in chosen time windows averaged across participants. B) Source reconstructed inflated brains of averaged beta activity in chosen time windows averaged across participants.

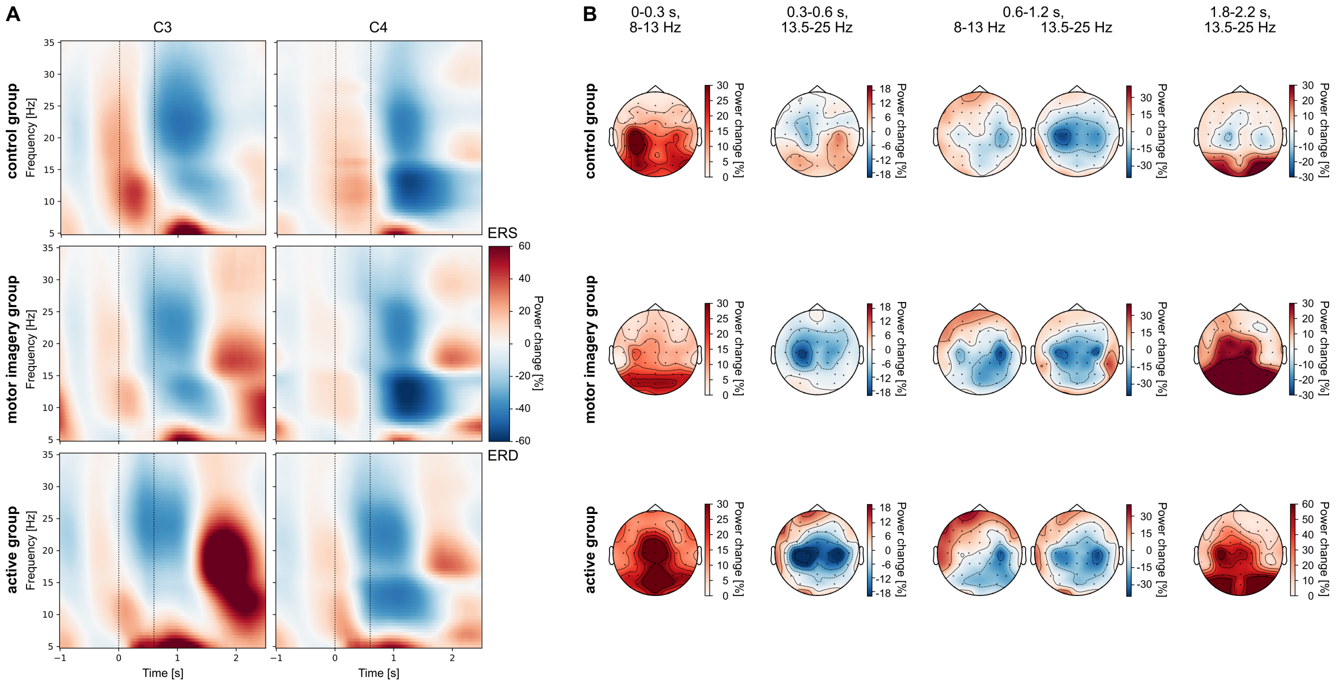

Fig. S3. EEG data reaching task. A) Time-frequency representation of grand averaged data per group in C3 and C4. Color represents power change in percent relative to the baseline window from -0.75 to -0.25s. The *go*-signal for the control group was at 0.6 s; for the MI group the *go*-signals for the imagined and overt reach were at 0 and 0.6 s, respectively; the active group started overt reaches after the signals at 0 and 0.6 s. $n_{\text{control group}}$ = 18, $n_{\text{MI group}}$ = 18, $n_{\text{active group}}$ = 16. B) Topoplots of averaged alpha and beta activity in chosen time windows averaged across participants in each group .

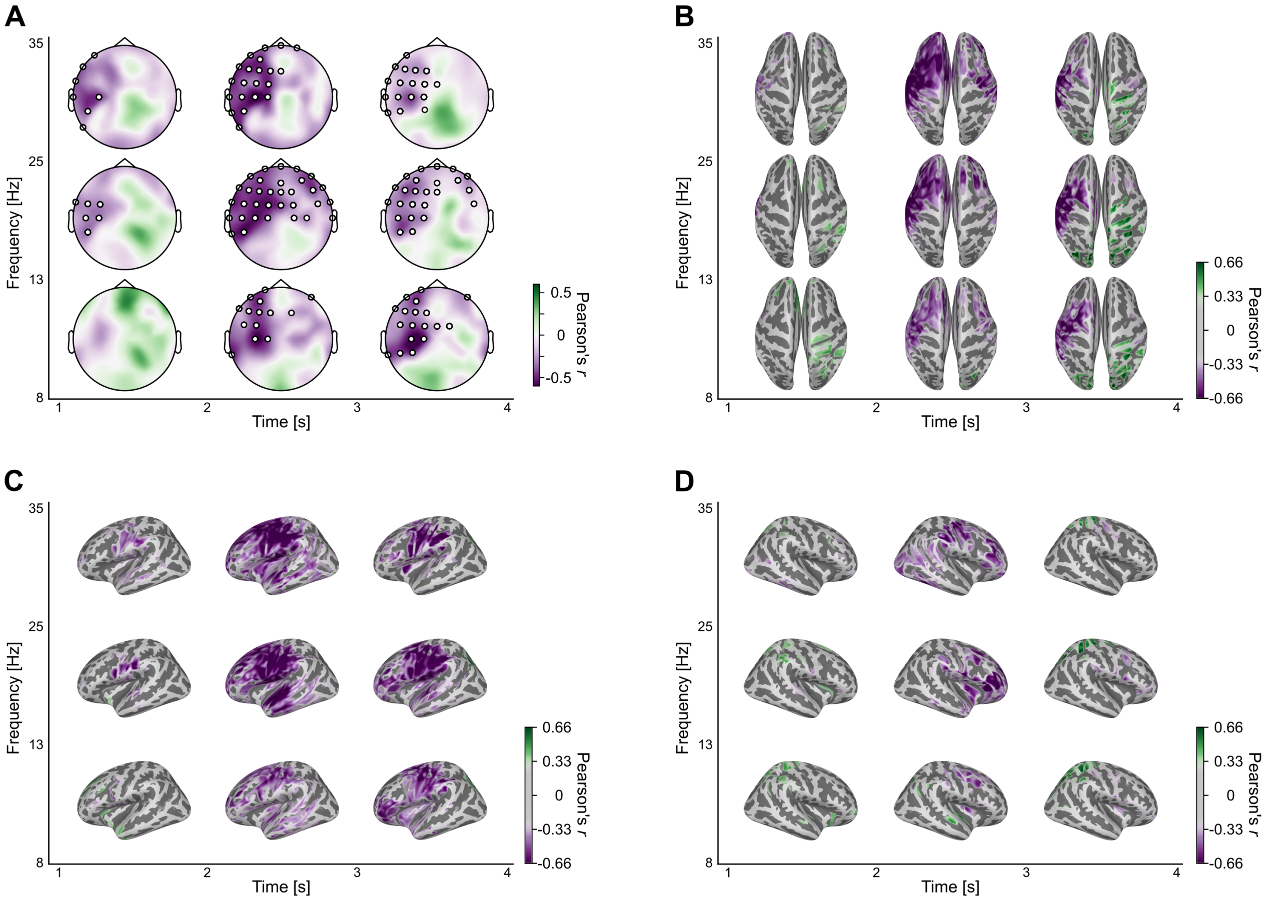

Fig. S4. Pearson's correlations of change of error in the reaching task with power change in the imagined fist clenching task across participants. A) Topoplots of different time windows and frequency ranges displaying the distribution of correlation values. Correlation values were averaged in the specified time and frequency ranges. Channels of the significant cluster are marked in white. B), C) & D) Inflated brains display correlation values for different time windows and frequency ranges in source space. Correlation values are averaged in the specified time and frequency ranges. The colorbars were maintained identical to those in Fig. 4D to facilitate comparison. B) Dorsal view. C) Lateral view left hemisphere. D) Lateral view right hemisphere.

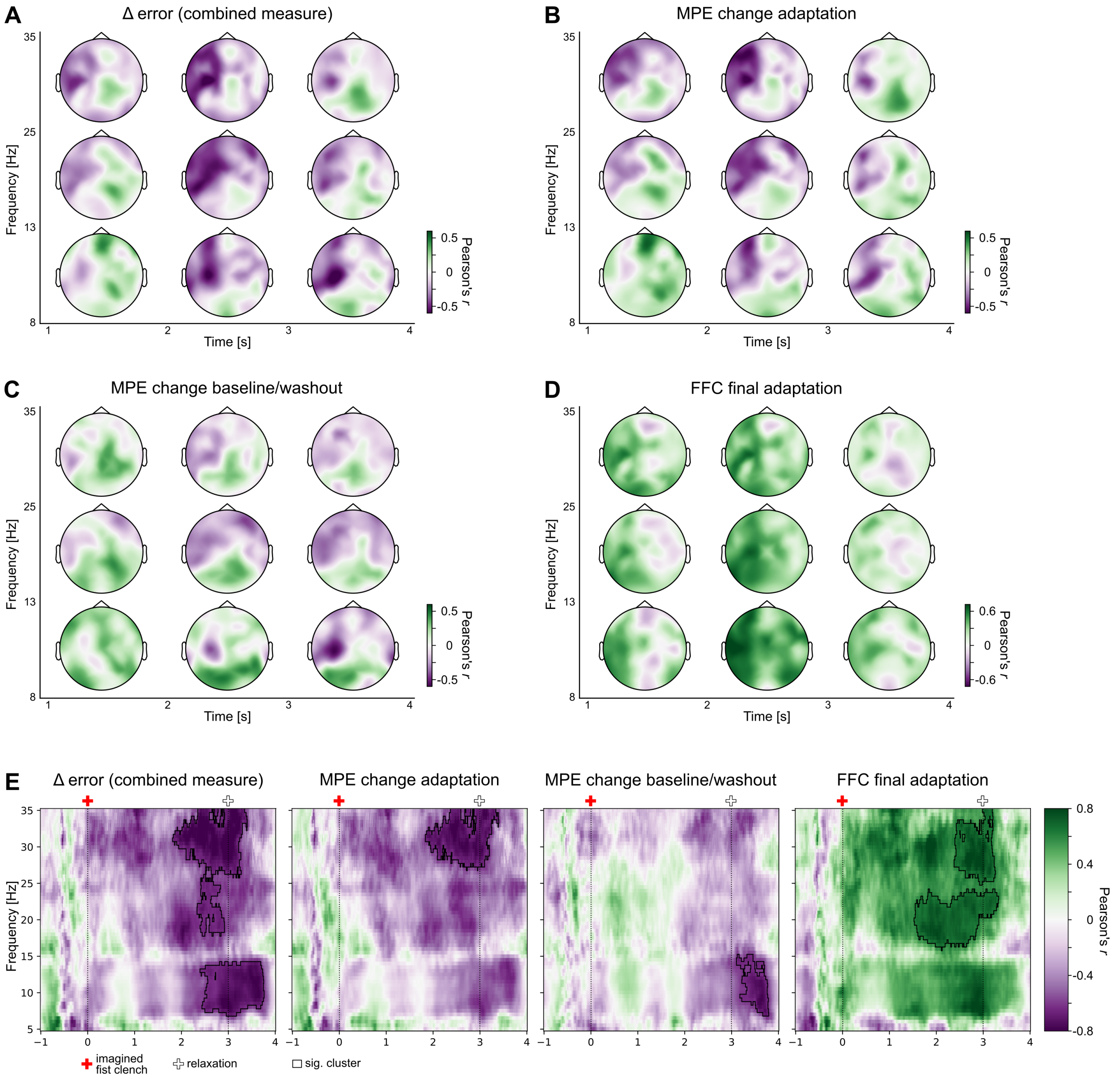

Fig. S5. Pearson's correlations of behavioral measures in the reaching task with power change in the imagined fist clenching task across participants. A - D) Topoplots of different time windows and frequency ranges displaying the distribution of correlation values. Correlation values were averaged in the specified time and frequency ranges. We have shown before that behavioral measures are strongly correlated in our specific setup of this task (2). A) Combined measure (change of error) for comparison (p_{cluster} = 0.023). B) Change of MPE from beginning to end of adaptation (p_{cluster} = 0.102). C) Change of MPE from baseline to washout (p_{cluster} = 0.585). D) Average FFC in the last 4 adaptation blocks. Note that here the inverse of the correlation pattern is expected, because the better the performance improvement, the bigger the FFC final adaptation measure (p_{cluster} = 0.004). E) Time-frequency plots of the correlation between the measure indicated on top of each respective plot with power change in the imagined fist clenching task across participants in C3. Cluster-based permutation tests within C3 (initial grouping threshold = 0.01, 1000 permutations) found the following significant clusters: Combined measure: p_{beta} = 0.003, p_{alpha} = 0.01; MPE change adaptation: p = 0.011; MPE change baseline/washout: p = 0.041; FFC final adaptation: p_{beta} = 0.008, $p_{\text{high beta}}$ = 0.017.

Table S1. Relationship between behavior and EEG measures. Pearson's correlation of change of error in the reaching task with peak power changes in the imagined fist clenching task across participants in C3 for different scenarios. ERS - maximal positive power change value in the alpha band (if not indicated differently) between 2 and 4s. ERD - maximal negative power change value in the alpha band (if not indicated differently) between 0 and 2s. Rebound - difference between peak ERS and peak ERD. All p-values are uncorrected. Significant correlations are in bold.

Change of error correlated with	ERS	ERD	Rebound
Exclusion of participant #51<br>(most influential for ERS correlation)	$r(13)$ = **-0.615**<br>p = 0.015	$r(13)$ = 0.156<br>p = 0.579	$r(13)$ = **-0.682**<br>p = 0.005
Exclusion of participant #29<br>(most influential for rebound correlation)	$r(13)$ = **-0.676**<br>p = 0.006	$r(13)$ = -0.282<br>p = 0.309	$r(13)$ = **-0.674**<br>p = 0.006
Baseline window -1 : 0<br>(instead of -0.75 : -0.25)	$r(14)$ = **-0.764**<br>$p < 0.001$	$r(14)$ = -0.122<br>p = 0.653	$r(14)$ = **-0.726**<br>p = 0.001
Average reference<br>(instead of current source density)	$r(14)$ = **-0.52**<br>p = 0.039	$r(14)$ = 0.136<br>p = 0.616	$r(14)$ = **-0.574**<br>p = 0.02
Morlet wavelet transformation<br>(instead of multitaper)	$r(14)$ = **-0.679**<br>p = 0.004	$r(14)$ = 0.078<br>p = 0.774	$r(14)$ = **-0.687**<br>p = 0.003
Beta band 13.5 - 25 Hz<br>(instead of alpha 8 - 13 Hz)	$r(14)$ = **-0.634**<br>p = 0.008	$r(14)$ = 0.046<br>p = 0.866	$r(14)$ = **-0.662**<br>p = 0.005
High beta band 25.5 - 35 Hz<br>(instead of alpha 8 - 13 Hz)	$r(14)$ = **-0.551**<br>p = 0.027	$r(14)$ = -0.391<br>p = 0.134	$r(14)$ = -0.354<br>p = 0.179

References

1. I Howard, JN Ingram, DW Franklin, DM Wolpert, Gone in 0.6 Seconds: The Encoding of Motor Memories Depends on Recent Sensorimotor States. *J. Neurosci.* **32**, 12756–12768 (2012).
2. M Gippert, et al., Prior Movement of One Arm Facilitates Motor Adaptation in the Other. *The J. Neurosci.* **43**, 4341–4351 (2023).
3. CR Harris, et al., Array programming with NumPy. *Nature* **585**, 357–362 (2020).
4. W McKinney, Data Structures for Statistical Computing in Python. (Austin, Texas), pp. 56–61 (2010).
5. P Virtanen, et al., SciPy 1.0: fundamental algorithms for scientific computing in Python. *Nat. Methods* **17**, 261–272 (2020).
6. F Pedregosa, Scikit-learn: Machine Learning in Python. *MACHINE LEARNING IN PYTHON* (2011).
7. JD Hunter, Matplotlib: A 2D Graphics Environment. *Comput. Sci. & Eng.* **9**, 90–95 (2007).
8. M Waskom, seaborn: statistical data visualization. *J. Open Source Softw.* **6**, 3021 (2021).
9. A Gramfort, MEG and EEG data analysis with MNE-Python. *Front. Neurosci.* **7** (2013).
10. I Winkler, S Debener, KR Muller, M Tangermann, On the influence of high-pass filtering on ICA-based artifact reduction in EEG-ERP in *2015 37th Annual International Conference of the IEEE Engineering in Medicine and Biology Society (EMBC).* (IEEE, Milan), pp. 4101–4105 (2015).
11. D Slepian, Prolate Spheroidal Wave Functions, Fourier Analysis, and Uncertainty-V: The Discrete Case. *Bell Syst. Tech. J.* **57**, 1371–1430 (1978).
12. A Gramfort, et al., MNE software for processing MEG and EEG data. *NeuroImage* **86**, 446–460 (2014).
13. FH Lin, et al., Assessing and improving the spatial accuracy in MEG source localization by depth-weighted minimum-norm estimates. *NeuroImage* **31**, 160–171 (2006).
14. VV Nikulin, FU Hohlefeld, AM Jacobs, G Curio, Quasi-movements: A novel motor–cognitive phenomenon. *Neuropsychologia* **46**, 727–742 (2008).
15. S Sporn, X Chen, JM Galea, The dissociable effects of reward on sequential motor behavior. *J. Neurophysiol.* **128**, 86–104 (2022).
16. M Gregg, C Hall, A Butler, The MIQ-RS: A Suitable Option for Examining Movement Imagery Ability. *Evidence-Based Complementary Altern. Medicine* **7**, 249–257 (2010).
